# ﻿New members of *Alternaria* (Pleosporales, Pleosporaceae) collected from Apiaceae in Algeria

**DOI:** 10.3897/mycokeys.113.138005

**Published:** 2025-02-04

**Authors:** Nabahat Bessadat, Nelly Bataillé-Simoneau, Justine Colou, Bruno Hamon, Kihal Mabrouk, Philippe Simoneau

**Affiliations:** 1 University of Angers, Institut Agro, INRAE, UMR IRHS, SFR QUASAV, 42 rue G. Morel, CS60057, 49071 Beaucouzé, France University of Angers Beaucouzé France; 2 Laboratory of Applied Microbiology, University Oran 1 Ahmed Ben Bella, BP1524 El M'naouer, Oran 31000, Algeria University Oran 1 Ahmed Ben Bella Oran Algeria

**Keywords:** *Daucuscarota*, *Embellisia*-like species, multi-gene phylogeny, new host record, new species, pathogenicity, taxonomy

## Abstract

*Alternaria* species have often been reported as plant pathogens and are commonly isolated from diseased plant hosts. During an investigation of this genus in Algeria, seven *Embellisia*-like isolates were collected from Apiaceae. Phylogenetic analysis using sequences at four loci, the internal transcribed spacer of the rDNA region (ITS), glyceraldehyde-3-phosphate dehydrogenase (*gpd*), translation elongation factor 1-alpha (tef1), and RNA polymerase second largest subunit (rpb2), revealed that these isolates grouped into three sections, namely *Embellisia*, *Embellisioides*, and *Eureka*. Four isolates had significant differences with their closest species and were determined to be new species, namely *Alternarialongiformis* and *A.radicicola***spp. nov.** The three other isolates of section Eureka were identified as *A.eureka* and *A.hungarica*, the latter species being described as a new record in Algeria. Detailed descriptions of new species are provided based on colony color, aspect, diameter, conidial size, septa, sporulation patterns and compared with other relevant *Alternaria* species within same sections. All these species were weakly pathogenic on carrot, coriander, and fennel under greenhouse experiments. Apiaceae may constitute a reservoir of *Alternaria* species that could represent potential pathogens for other plant families.

## ﻿Introduction

*Alternaria* is a large, pleomorphic genus in Pleosporaceae, whose taxonomy has strongly evolved in recent years. *Alternariatenuis* (the synonym of *A.alternata*), the type of the genus, was introduced by Nees (1816) as harboring muriform and catenulate conidia. Since then, many new species were proposed in the genus (He et al. 2024; Huang et al. 2024; Nwe et al. 2024). Currently, more than 850 species epithets are listed in Index Fungorum (http://www.indexfungorum.org/; accessed 04 December 2024), but only 360 of them are recognized as different species, although not all of them have been subjected to molecular phylogenetic studies (Wijayawardene et al. 2020). This number is continuously growing after new discoveries (Li et al. 2022, 2023). The genus includes plant pathogens that cause a variety of important crop plant diseases (Simmons 2007; Haituk et al. 2023; Cao et al. 2024) as well as opportunistic species causing human infections (Pastor and Guarro 2008; Kim et al. 2022). It also includes numerous saprobic species that cause post-harvest rots of agricultural products (Thomma 2003; Lee et al. 2015) and decay of organic matter in natural ecosystems (Song et al. 2010; Mollea and Bosco 2020).

Hundreds of descriptions were previously made by Simmons (2007), who introduced standards to get unified taxonomic concepts on *Alternaria* species based on the shape, size, and septation of conidia, as well as sporulation patterns. However, the identification remained challenging due to the impact of environmental conditions and intra-specific variations. In recent years, several studies using phylogenetic analyses based on 3–8 conserved protein-coding genes helped to clarify the taxonomic placement of a large number of species into sections and allowed the establishment of several re-descriptions (Lawrence et al. 2012; Woudenberg et al. 2013; Liu et al. 2016; Al Ghafri et al. 2019; Marin Felix et al. 2019; Gannibal et al. 2022; Li et al. 2023).

The genus is now separated into 29 sections and seven monotypic lineages based on molecular and morphological data (Li et al. 2023) and accommodates all former alternarioid hyphomycetes (13 genera), including the type species of the genus *Embellisia*.

*Embellisia* has been described in order to separate atypical taxa, including species of *Helminthosporium* (Simmons 1971). *Embellisia*-like species are characterized mainly by darkly pigmented, multicelled conidia, which are typically dictyosporous or phragmosporous, arranged singly or in chains on conidiophores (Simmons 1990; Woudenberg et al. 2013; Li et al. 2023); on mycelium, chlamydospores are often formed (Simmons 1971, 1983). One species of *Embellisia* had a teleomorphic state obtained in axenic culture. The teleomorph, *Allewia*, was segregated from the *Lewia*/*Alternaria* relationship only as a state of *Embellisia* (Simmons 1990, 2007). Research using molecular methods revealed that *Embellisia* does not form a monophyletic genus (Lawrence et al. 2012, 2013) and that the polyphyletic nature of this group included 24 species (Woudenberg et al. 2013; Gannibal and Gasich 2019). Most of the *Embellisia*-like species have been moved to genus *Alternaria* and were divided into four groups (Lawrence et al. 2012) that were later elevated to section status by Woudenberg et al. (2013). The AlternariasectionEmbellisia contains three species, including the former type species (*Embellisiaallii*) of the genus *Embellisia*. This section and section Embellisioides were identical to *Embellisia* group I and III mentioned by Lawrence et al. (2012), respectively. Two species of the *Embellisia* group, II and IV, were moved to sections *Phragmosporae* and *Eureka*, respectively. Species from groups II and IV displaying morphological characters different from *Embellisia* were attributed to *Alternaria* sections based on the oldest species residing in the respective sections (i.e., sections *Brassicicola* and *Cheiranthus*). One species (*E.dennissii*) did not cluster within any Alternaria section and forms a separate lineage (Woudenberg et al. 2013). Another *Embellisia* species (*E.astragali*) was later placed in section Undifilum and renamed as *A.gansuense* (Liu et al. 2016). Finally, one species (*E.annulata*) was moved to the genus *Paradendryphiella*. Many species of sections *Embellisia* and *Eureka* constitute under-sampled and poorly described taxa. For example, *A.hungarica* was not included in the revision of the genus *Alternaria* (Woudenberg et al. 2013) since only the ITS sequence was available in the database (Toth et al. 2011).

Apiaceae is a wide botanical plant family that is known to harbor several pathogenic and opportunistic *Alternaria* spp. (Bulajić et al. 2009; Nishikawa and Nakashima 2020; He et al. 2021). During the investigation of *Alternaria* species in Algeria, strains with morphological characters typical of *Embellisia*-like species were isolated from wild and cultivated Apiaceae plants. The study was designed to determine their identity based on a polyphasic approach including morphology, phylogenetic analyses, and pathogenicity testing.

## ﻿Materials and methods

### ﻿Isolation

Fresh samples of infected tissue (leaves, roots) were collected from Apiaceae in north-west regions of Algeria (Mostaganem, Oran, Mascara) during the 2020 and 2021 growing seasons. Samples were placed in paper bags and taken to the laboratory for further examination and isolation. Fungi were isolated by cutting one or two fragments (5–10 mm^2^) per necrotic tissue from the margin of lesions and surface-disinfected by immersing in 2% sodium hypochlorite solution for 2 min. Samples were then rinsed in sterile distilled water twice, dried with sterilized paper towels, and placed on potato carrot agar (PCA) (Simmons 2007). All the plates were incubated at room temperature under natural daylight for 4–6 days. Hyphae were picked out of the periphery of the colonies and inoculated onto new PCA plates. Following 1–2 weeks of incubation, the monoconidial culture method was performed to obtain pure cultures. All fungal strains were stored in 30% sterilized glycerol at -80 °C in the COMIC collection (Angers University, France) and potato dextrose agar (PDA) slants at 4 °C for further studies. The holotype specimen of the new species is preserved in a metabolically inactive state via deep-freezing at the INH herbarium (using the COMIC technical platform and the infrastructures of the CFBP, a culture collection recognized under the International Code of Nomenclature of Procaryotes (Oren et al. 2023). The Ex-type strains are preserved in the Collection of the Westerdijk Fungal Biodiversity Institute (CBS), Utrecht, the Netherlands.

### ﻿Morphological studies

Morphological observation for *Alternaria* description was based on macroscopic (color, aspect, and diameter of the colonies) and microscopic (microstructure) characters according to Simmons (2007) and Marin-Felix et al. (2019). Vegetative and developmental structures were mounted in 100% lactic acid from colonies sporulating on PCA medium at room temperature (25–28 °C) under natural daylight. Conidia mean length and width values were calculated for each isolate from 50 measurements of randomly selected conidia and conidiophores. In addition, the number of transverse septa and longitudinal septa were determined, as well as the maximum number of septa per isolate. Immature conidia (i.e., lacking septation and pigmentation) were not considered. Conidial germination was observed from conidia placed in sterilized distilled water drops on slides and covered with glass covers for 24–48 h. The slides were kept in Petri dishes containing filter papers soaked with 10 mL of water to prevent the conidia from drying out. Germ tubes and mycelia were observed with a microscope (Optika 190B, Italy) at 400×. For cultural characterization, strains were grown on 90 mm diameter PDA, malt extract agar (MEA), oatmeal agar (OA), and PCA Petri dishes for 7 days at 25 °C. Mycelial agar discs, 5 mm in diameter, were cut from vegetative areas of 7-day-old PDA cultures grown at 25 °C. Single discs were placed onto each medium. Color notations were rated according to the color charts of Kornerup and Wanscher (1978). For new species, plates were incubated at 4, 16, 20, 25, 30, 35, and 40 °C in the dark for 7 days. Colony diameters were then measured. Each inoculation experiment was done in triplicate. Living cultures were deposited in the Westerdijk Institute Culture Collection (CBS). Taxonomic information of new taxa was submitted to MycoBank (http://www.mycobank.org). Reference isolates of *A.hungarica* (CBS 123925) (Toth et al. 2011) and another strain previously isolated from Solanaceae (NB354) described initially as *A.lolii* (Bessadat et al. 2021) were used as standards for the discrimination of the new isolates.

### ﻿DNA extraction, PCR amplification, and sequencing

Genomic DNA was extracted from fungal mycelia grown on PDA for 5–7 days, using lysis buffer (50 mM Tris-HCl pH 7.5, 50 mM EDTA, 3% SDS, 1% 2-mercaptoethanol) and the miniprep (microwave) method described in Goodwin and Lee (1993). For accurate identification of the isolates, DNA amplification of portions of the glyceraldehyde-3-phosphate dehydrogenase (*gpd*), translation elongation factor 1-alpha (*tef1*), and RNA polymerase second largest subunit (*rpb2*) genes was performed using the primer pairs gpd1–gpd2 (Berbee et al. 1999), EF1–728F/EF1–986R (Carbone and Kohn 1999), and RPB2–5F/RPB2–7cR (Liu et al. 1999), respectively. rDNA ITS was amplified using ITS1/ITS4 primers (White et al. 1990). A total of 50 μL of a PCR reaction mixture containing 75 mM Tris-HCl pH 9.0, 20 mM (NH_4_)2SO_4_, 0.01% (w/v) Tween 20, 1.5 mM MgCl_2_, 200 µM of each deoxyribonucleotide triphosphate, 1 unit of thermostable DNA polymerase (GoTaq, Promega), and 400 nM of each relevant oligonucleotide primer. The thermocycling parameters consisted of an initial denaturation step of 5 min at 94 °C followed by 35 cycles of 30 s at 94 °C, 30 s at 48 °C, and 90 s at 72 °C for *gpd* and ITS, and 40 cycles of 30 s at 94 °C, 30 s at 59 °C, and 45 s at 72 °C for *tef1*, and a final elongation step of 7 min at 72 °C. The partial *rpb2* gene was obtained using a PCR protocol of 5 cycles of 45 s at 94 °C, 45 s at 60 °C, and 2 min at 72 °C, followed by 5 cycles with a 58 °C annealing temperature and 30 cycles with a 54 °C annealing temperature. DNA amplification products were analyzed by electrophoresis in 1.2% (w/v) agarose in 0.5 × TAE buffer. Successful products were sequenced by GATC company (Germany), and sequences were deposited at the international GenBank database (https://www.ncbi.nlm.nih.gov/) (Table 1).

**Table 1.** Strain origins and GenBank accession numbers of DNA sequences used in this study.

Section	Species	Strain	Host/Substrate	Country	GenBank accession numbers*				Reference
					ITS	*gpd*	*rpb2*	*tef1*	
*Embellisia*	*A.chlamydosporigena*	CBS 341.71	air	USA	KC584231	KC584156	KC584451	KC584710	Woudenberg et al. (2013)
	*A.embellisia*	CBS 339.71	*Alliumsativum*	USA	KC584230	KC584155	KC584449	KC584708	Woudenberg et al. (2013)
	***A.radicicola* sp. nov.**	**NB794**	***Daucuscarota***	**Algeria**	**OR085519**	**OP297089**	**OP320886**	**OP320892**	Present study
	***A.radicicola* sp. nov.**	**NB830^T^**	***Daucuscarota***	**Algeria**	**OR085521**	**OP297090**	**OP320887**	**OP320893**	Present study
	***A.radicicola* sp. nov.**	**NB936**	***Daucuscarota***	**Algeria**	**OR085524**	**OR099832**	**OR099834**	**OR099836**	Present study
	*A.tellustris*	CBS 538.83^T^	soil	USA	FJ357316	AY562419	KC584465	KC584724	Woudenberg et al. (2013)
*Embellisioides*	*A.botryospora*	CBS 478.90^t^	*Leptinelladioica*	New Zealand	AY278844	AY278831	KC584461	KC584720	Woudenberg et al. (2013)
	*A.hyacinthi*	CBS 416.71^T^	*Hyacinthusorientalis*	Netherlands	KC584233	KC584158	KC584457	KC584716	Woudenberg et al. (2013)
	*A.lolii*	CBS 115266^T^	*Loliumperenne*	New Zealand	JN383492	JN383473	KC584460	KC584719	Woudenberg et al. (2013)
	***A.longiformis* sp. nov.**	**NB354^T^**	**Tomato leaf**	**Algeria**	**OK353787**	MK904520	MK904534	MK904543	Bessadat et al. (2021)
	***A.longiformis* sp. nov.**	**NB930**	***Daucuscarota***	**Algeria**	**OR085523**	**OP297094**	**OP320891**	**OP320897**	Present study
	*A.planifunda*	CBS 537.83^T^	*Titricumaestivum*	Australia	FJ357315	FJ357303	KC584463	KC584722	Woudenberg et al. (2013)
	*A.proteae*	CBS 475.90^T^	Protea	Australia	AY278842	KC584161	KC584464	KC584723	Woudenberg et al. (2013)
	*A.tumida*	CBS 539.83^T^	*Titricumaestivum*	Australia	FJ266481	FJ266493	KC584466	KC584725	Woudenberg et al. (2013)
*Eureka*	*A.anigozanthi*	CBS 121920^T^	*Anigozanthus* sp.	Australia	KC584180	KC584097	KC584376	KC584635	Woudenberg et al. (2013)
	*A.cumini*	CBS 121329^T^	*Cuminumcyminum*	India	KC584191	KC584110	KC584391	KC584650	Woudenberg et al. (2013)
	*A.eureka*	CBS 193.86^T^	*Medicagorugosa*	Australia	JN383490	JN383471	KC584456	KC584715	Woudenberg et al. (2013)
	***A.eureka***	**NB968**	***Daucuscarota***	**Algeria**	**OR085525**	**OP297093**	**OP320890**	**OP320896**	Present study
	*A.geniostomatis*	CBS 118701^T^	*Geniostoma sp*.	New Zealand	KC584198	KC584117	KC584400	KC584659	Woudenberg et al. (2013)
	*A.hungarica*	CBS123925^T^	Wheat	Hungary	NR135944	**OR099833**	**OR099835**	MF070326	Toth et al. (2011)
	***A.hungarica***	**NB803**	***Daucuscarota***	**Algeria**	**OR085520**	**OP297091**	**OP320888**	**OP320894**	Present study
	***A.hungarica***	**NB898**	***Daucuscarota***	**Algeria**	**OR085522**	**OP297092**	**OP320889**	**OP320895**	Present study
	*A.leptinellae*	CBS 477.90^T^	*Leptinelladioica*	New Zealand	KC584235	KC584160	KC584459	KC584718	Woudenberg et al. (2013)
	*A.triglochinicola*	CBS 119676^T^	*Triglochinprocera*	Australia	KC584222	KC584145	KC584437	KC584695	Woudenberg et al. (2013)
Outgroup	*Paradendryphiellasalina*	CBS 302.84^T^	*Cancer pagurus*	North Sea, Skagerrak	JN383486	JN383467	KC484450	KC584709	Woudenberg et al. (2013)

Notes: Accession numbers in bold correspond to sequences generated in the present study.

### ﻿Phylogenetic analysis

DNA sequences from isolates and strains of related species retrieved from GenBank were concatenated and aligned by the MUSCLE algorithm using MEGA 7 (Kumar et al. 2016). Phylogenetic analysis was done using the maximum likelihood (ML) and Bayesian inference (BI) approaches under IQTree v.1.6. (Nguyen et al. 2015) and MrBayes v.3.2.1 (Ronquist et al. 2012), respectively. The best-fit evolutionary model (TNe+R2) was calculated by ModelFinder (Kalyaanamoorthy et al. 2017) under the Bayesian Information Criterion (BIC) selection procedure. The ML analysis was carried out with 1000 ultrafast bootstrap replicates, and only values above 95% were considered significant. BI was performed to estimate the posterior probabilities (PP) of tree topologies based on the Markov Chain Monte Carlo (MCMC) analysis with four chains (Huelsenbeck and Ronquist 2001; Zhaxybayeva and Gogarten 2002; Ronquist et al. 2012), 1M generations, sampled every 1000 generations. Burn-in was set to 25%, and only PP values above 0.95 were considered significant.

### ﻿Pathogenicity test

Pathogenicity experiments were conducted using carrot (var. Muscade), fennel (var. Latina), and coriander. Seeds were sown in plastic pots (8 cm in diameter by 12 cm deep) containing an autoclaved potting soil: sand mixture (3:1) in a greenhouse at 25 ± 5 °C. The pots were incubated for 8 weeks in the greenhouse prior to inoculation (Bessadat et al. 2016, 2021). Each fungal isolate (Table 1) was grown on PCA for 14 days under natural daylight. Conidia were removed from the surface of fungal colonies with sterilized distilled water containing 0.01% Tween 80; the spore suspensions were adjusted to 10^4^ conidia/mL (Bessadat et al. 2016, 2019). The potted Apiaceae plants were inoculated by spraying a spore suspension of each isolate on the foliage, and the pots were covered with polyethylene bags for 2 days to maintain a high level of humidity. Negative control plants were sprayed with distilled water alone. The percentage of leaf surface covered with necrotic lesions was estimated for each plant (first three leaves per plant) after 14 days of inoculation. Each inoculation experiment was done in triplicate. Mean percentage of leaf necrotic area (l. n. a.) was determined. Statistical analyses of the normalized data were conducted using ANOVA and Tukey’s post-hoc tests with R software (R4.3.1) (Faraway 2002).

## ﻿Results

### ﻿Phylogenetic analyses

**Figure 1.**
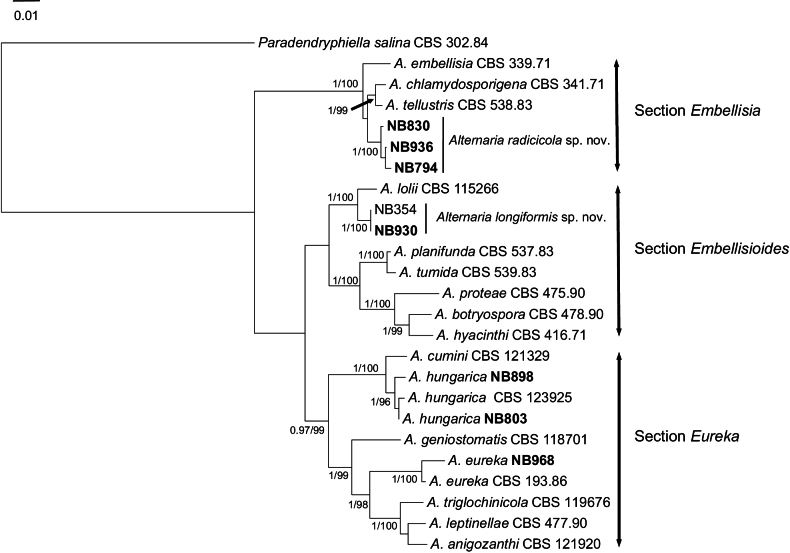
Phylogenetic tree constructed by the maximum likelihood method from the alignment of ITS, *gpd*, *rpb2*, and *tef1* of *Embellisia*-like isolates of *Alternaria*. Bootstrap support values greater than 95% and Bayesian posterior probabilities greater than 0.95 are indicated near nodes. Strains collected on Apiaceae are in bold characters.

Morphological observation of isolates from Apiaceae collection revealed that seven produced conidia with typical characteristics reported for *Embellisia*-like species. Phylogenetic analysis of *gpd* sequences from these isolates and corresponding sequences from strains representative of the 29 sections defined in the genus *Alternaria* confirmed that they all belong to sections *Embellisia*, *Embellisioides*, and *Eureka* (Suppl. material 1); these three sections group species formerly assigned to the genus *Embellisia*. Further identification was obtained through multilocus phylogenetic analysis with concatenated sequences of ITS, *gpd*, *rpb2*, and *tef1* from 24 strains, including 16 references from all recognized species of sections *Embellisia*, *Embellisioides*, and *Eureka*. Besides sequences of the seven isolates from Apiaceae, the analysis included sequences of one strain (NB354) formerly isolated from Solanaceae and identified as belonging to section Embellisioides. Sequences of *Paradendryphiellasalina* (CBS 302.84) were used as an outgroup. The combined dataset contained a total of 2028 characters with gaps, and 352 were parsimony-informative. Tree topologies computed from the BI and ML analyses were similar, and the ML tree was shown in Fig. 1.

The results indicated that three isolates (NB794, NB830, NB936) fell into the section Embellisia, forming a distinct clade with high support (PP/BS values of 1/100%) close to *A.chlamydosporigena* and *A.tellustris* and were considered as a new species named *A.radicicola* sp. nov. The results also showed that one isolate (NB930) clustered with a strain formerly isolated from tomato (NB354) in a distinct clade (PP/BS values of 1/100%) among other species from Embellisioides. These two strains, closely related to section *A.lolii*, were also considered as belonging to a newly described species named *A.longiformis* sp. nov. The phylogenetic analysis showed that three remaining isolates from Apiaceae grouped within section Eureka, where they formed two different clades: one (NB968) with *A.eureka*CBS 193.86 (PP/BS values 1/100%) and one (NB803, NB898) with *A.hungarica* (CBS 123925) related to but distinct (PP/BS values 1/100%) from *A.cumini* (CBS 121329).

### ﻿Taxonomy

#### Section ﻿Eureka

##### ﻿ Alternaria eureka

Taxon classificationFungiPleosporalesPleosporaceae

E.G. Simmons

9A5533D8-437B-5E06-A0A2-25E868E4F586

Fig. 2

≡ Embellisiaeureka (E.G. Simmons) E.G. Simmons, Mycotaxon 38: 260. 1990.
= Lewiaeureka E.G. Simmons, Mycotaxon 25: 304. 1986.
≡ Allewiaeureka (E.G. Simmons) E.G. Simmons, Mycotaxon 38: 264. 1990.

###### Specimen examined.

Algeria • Ain Témouchent City, Chabaat El Lahame, from leaves of wild *Daucuscarota*. 08 April, 2021, N. Bessadat, Living culture NB968.

###### Description.

**Figure 2.**
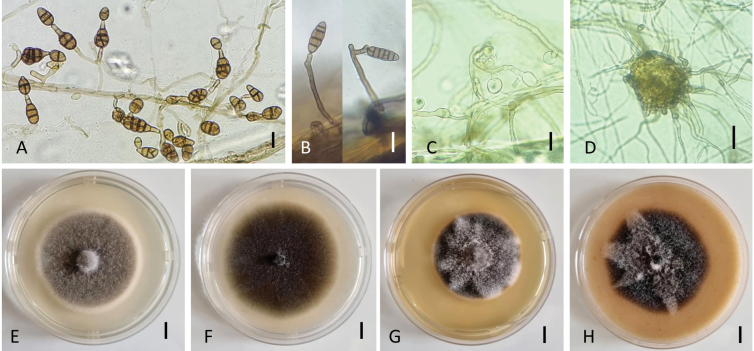
Morphology of *Alternariaeureka* (strain: NB968). Conidiophores and conidia on PCA for 7 days at 22 °C (**A**) and on inoculated carrot leaves after 24 days (**B**); Chlamydospores (**C**) and protoascomata (**D**) on PCA for 18 days at 22 °C. Colony phenotypes on PDA (**E**), PCA (**F**), MEA (**G**), and OA (**H**) for 7 days at 25 °C. Scale bar: 25 µm (**A–D**); 10 mm (**E–H**).

Colonies on PCA velvety to cottony colonies, mostly mycelial, subhyaline, loosely wooly, reaching 75 mm in diameter after 7 days; meager sporulation or lacking until hyphae are disturbed or scarified. Conidiophores emerging from the surface of agar or aerial vegetative hyphae scattered or clustered on cut agar in light-exposed areas. Primary conidiophores mostly simple, rarely branched, 25–75 × 5–6 µm, geniculate with 2–3(–4) conidiogenous loci. Secondary conidiophores, short, 3–7 × 3 µm, 1–2-celled, formed apically or sometimes laterally from primary conidia, mainly with one conidiogenous locus. Further geniculate extensions and conidium production yield several clusters of sporulation at the colony center. Sporulation pattern in single clumps of a few short branching chains consisting of 5–7 conidia. A high percentage of conidia solitary at any age of growth. Conidia ellipsoid to ovoid, with a rounded base and tapered apex; multiple transverse and longitudinal septa, conspicuously constricted near 2 or 3 transverse septa, in short chains. Mature conidia with 2–3 transverse septa and 0–3 longitudinal septa located mainly at the center of the colony, 20–24 × 8–15 µm; slightly older conidia, 25–34(–48) × 11–16 (–22) µm with 3–4(–5) transverse septa and 1–3 oblique or longitudinal septa. Conidial body color brown with relatively dark and thick transverse septa (Fig. 2A, B); the outer wall smooth or punctate. Formation of protoascomata and chlamydospores in the surface of vegetative mycelium conspicuous after 2–3 weeks of incubation. Chlamydospores hyaline, spherical or ovoid, terminal, sub-terminal or intercalary, measuring 13.5–22.5 × 10–15 µm, mostly single-celled (Fig. 2C). Protoascomata subspherical to ovoid, light brown with numerous outgrowths of hyphae, measuring 40–58 µm in diameter (Fig. 2D).

Culture characteristics at 25 °C in 7d—Colonies on PDA cottony, grayish yellow to yellowish grey (3C3/3D2) with white regular margins, attaining 60.5 ± 1.1 mm diam. (Fig. 2 E); reverse olive brown to yellowish brown (4F6/5F5). On PCA, velvety to glabrous, olive brown (4E5), 67.4 ± 0.4 mm diam. (Fig. 2F); reverse olive brown (4E4). On MEA, cottony, dull green (28D3) with a greenish-grey surface (28C2) and white regular margins, 51.9 ± 0.7 mm diam. (Fig. 2G), reverse yellowish brown to brownish orange (5F5/5C4). On OA, velvety with a cottony center, yellowish brown to dark blond (5E4/5D4), 60.6 ± 1.5 mm diam. (Fig. 2H); reverse brownish grey to yellowish brown (5F2/5E4). Sporulation of NB968 isolate occurred mainly in PCA and PDA after 7 days of incubation. It was poor on MEA and OA.

###### Notes.

The multilocus phylogeny revealed that NB968 significantly clustered with *A.eureka* (ex-type, CBS 193.86), both forming a branch separated from other species in section Eureka. Cultural characters, sporulation patterns, and conidia shapes of *A.eureka* are different from other members of section Eureka. A high percentage of conidia have conical apical cells but differ in size and number of transverse septa from *A.hungarica* (15–30 × 12–13 vs. 25–48 × 8–22, respectively). Conidiogenous axes, conidiophores, and conidia chains of the latter species are longer than those of *A.eureka* (30–352 × 3.8–5 µm vs. 25–75 × 5–6 µm, respectively). Conidia chain formation of *A.eureka* is rare, while in *A.hungarica*, short-branched chains of 3–4 units are conspicuous. *Alternariaeureka* and *A.hungarica* conidia consist of numerous longitudinal and/or oblique septa forming multi-celled segments; the two species are, however, distinct from each other according to the sporulation pattern (see *A.hungarica* notes). *Alternariaeureka* is the only species from section Eureka known to have a sexual state (Simmons 1986); it also produces hyaline chlamydospores and sclerotia upon aging, which are lacking in *A.hungarica* and *A.cumini*.

##### ﻿ Alternaria hungarica

Taxon classificationFungiPleosporalesPleosporaceae

B. Toth, J. Varga, M. Csosz, E.G. Simmons & R.A. Samson

144B262A-C77D-5A58-B6EF-83D161DD6E43

Fig. 3

###### Specimen examined.

Algeria • Mostaganem City, Kheir Eddine, from leaves of cultivated *Daucuscarota*. 01 December, 2020, N. Bessadat, Living culture NB968; ibid. Algeria, Ain Témouchent province, Bouzedjar, from leaves of wild *Daucuscarota*. 02 February, 2020, N. Bessadat, Living culture NB803.

###### Description.

**Figure 3.**
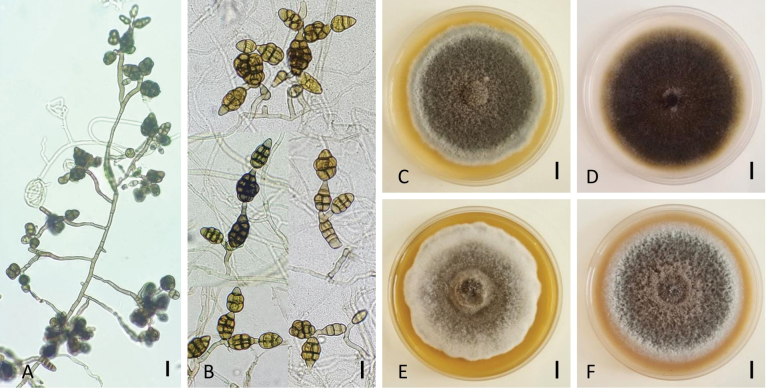
Morphology of *Alternariahungarica* (strain: NB803). Sporulation patterns on PCA for 14 days at 22 °C (**A**); Conidiophores and conidia on PCA for 7 days at 22 °C (**B**); Colony phenotypes on PDA (**C**), PCA (**D**), MEA (**E**), and OA (**F**) for 7 days at 25 °C. Scale bar: 25 µm (**A, B**); 10 mm (**C–F**).

Colonies on PCA velvety, approximately 70 mm in diameter, with three discrete concentric rings of growth after 7 days. Aerial axes abundant and reaching a size length of 113–387 µm, with 5 to 10 lateral conidiogenous branches and tips in light-exposed zones of young parts of the colony (Fig. 3A). These branches most frequently on the upper portions of aerial conidiogenous elements, forming an arachnoid layer of branching hyphae. Primary conidiophores formed outside the center of the colony, simple, rarely branched, produced at the substrate surface, conspicuous, straight, or geniculate, up to 30–110(–352) × 3.8–5 µm, often developing through geniculate extensions. Each conidiophore bearing 1–3 conidia, sometimes with additional short chains of 2–3 conidia through secondary conidiophores formed mainly from distal terminal conidial cells (Fig. 3B). Secondary conidiophores short, 20–52(–105) × 3–5 µm, with one or two conidiogenous sites. A high percentage of conidia of any age remaining solitary. The sporulation patterns in small clumps on elongate-branched sporulating hyphae forming a brown layer at the center of the colony. Conidia medium brown to brown, short ellipsoid, ovoid, muriform, mainly beakless or with a rounded apex (Fig. 3B). Dominant size range: 22–37(–40) µm long × 13–20 μm width with 2–4(–5) transverse septa and 0–2(–3) longitudinal septa in each of the transverse segments. In the center of the colony, a high percentage of dark pigmented conidia mature of smaller size, 17.5–25 × 12–17.5 µm, initiating chain development. All maturing conidia distinctly constricted at their transverse septa, developing thick and dark-brown pigmented outer walls and sometimes with a punctate surface.

Culture characteristics at 25 °C in 7d—Colonies on PDA cottony compact, greyish green (30E5/30E7) with irregular margins, attaining 67.5 ± 0.6 mm diam. (Fig. 3C); reverse olive (3E4). On PCA, velvety, olive brown (4E4), 69.4 ± 0.5 mm diam. (Fig. 3D); reverse grayish same color. On MEA, cottony, olive (1E3) with a grayish surface, irregular margins, 65.1 ± 0.6 mm diam. (Fig. 3E); reverse olive brown (4D3). On OA, cottony, grey to dull green (29D4) with a grayish surface, 63.8 ± 1.0 mm diam. (Fig. 3F); reverse olive brown (4E3). Sporulation of studied isolates occurs in all tested media after 7 days of incubation. It was abundant on PCA and PDA but moderate on MEA and OA.

###### Notes.

NB803 and NB898 formed a well-supported clade with *A.hungarica* (ex-type, CBS 123925) phylogenetically close to *A.cumini* (ex-type, CBS 121329). Between these three strains and *A.cumini* (ex-type, CBS 121329), there were 2/464 differences in ITS, 9/529 in *gpd*, 10/833 in *rpb2*, and 2/199 in *tef1*. Morphological features of two isolates from carrot leaves (wild and cultivated) are similar to *A.hungarica* and did not contradict descriptions of Toth et al. (2011). The conidial and sporulation characteristics resemble those of CBS 123925 but with some variations in cultural characteristics (colony color and texture on PDA). Isolates NB803 and NB898 form a less dense mycelium but are darker than CBS 123925. Cultural characters, sporulation pattern, and conidia shape of this species are different from *A.cumini* (17.5–40 × 12–20 vs. 50–90 × 13–23 μm, respectively). The latter species produce short and erect conidiophores of 18–60 × 5–7 μm (Nishikawa and Nakashima 2020), while *A.hungarica* form long geniculate conidiophores (30–352 × 3.8–5 µm). A high percentage of conidia are in clumps or short chains in *A.hungarica*, while *A.cumini* produces conidia in simple chains. Both species produce dark yellow (4C8) pigmentation on PDA after 7 days, which is lacking in *A.eureka*.

#### ﻿Section Embellisia

##### ﻿ Alternaria radicicola

Taxon classificationFungiPleosporalesPleosporaceae

N. Bessadat & P. Simoneau sp. nov.

A6F8A7F5-8031-5CBE-9FA5-C0503522037D

 848584

Fig. 4

###### Etymology.

Name refers to the organ from which the species was isolated, carrot roots.

###### Type.

Algeria • Oran market on infected roots of *Daucuscarota*. 16 July, 2020, N. Bessadat, (INH001054, holotype), preserved in a metabolically inactive state via deep freezing at INH herbarium, France, using the COMIC technical platform, ex-type cultures (CBS 149902, NB830).

###### Description.

**Figure 4.**
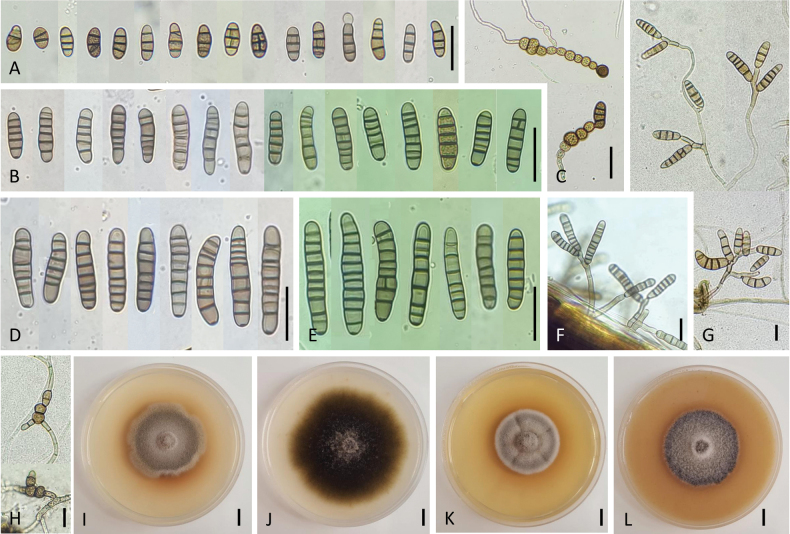
Morphology of *Alternariaradicicola* sp. nov. from *Daucuscarota*: Conidia on PCA for 7 days at 22 °C (**A, B, D**); Chlamydospores from germinating conidia (**C**); Conidia and conidiophores on inoculated coriander leaves at 7 DAI (**E, F**); Sporulation patterns on PCA for 14 days at 22 °C (**G**); Chlamydospores in the surface of vegetative mycelium (**H**); Colony phenotypes on PDA (**I**), PCA (**J**), MEA (**K**), and OA (**L**) for 7 days at 25 °C. Scale bar: 25 µm (**A–H**); 10 mm (**I–L**).

On PCA, attaining 75 mm in diameter, velvety (NB830, NB936) to flat (NB794), sometimes with granular appearance by the presence of abundant intra-hyphal, dematiaceous, thick-walled chlamydospores after 8–14 days. Aerial mycelium sparse and submerged hyphae abundant, producing chlamydospores in culture formed from fertile hyphae with 3–5 transverse septa and sometimes one longitudinal. These fructifying elements arising from a distinct radial system of hyphae or near the substrate surface. Conidiophores arising directly from lateral and apical aerial axes, simple, septate, 20–30 × 3.5–5 μm, straight or geniculate at successive sites of conidium production, size of cells decreasing towards apex, rarely branched, cell walls thicker than those of vegetative hyphae, pale brown to brown in color, sometimes swollen at the base, rarely from chlamydospores. Conidiogenous branches, formed on fertile hyphae, 1–2-celled, 15–20 × 3 µm; the longest conidiophores reaching 31–88 μm in length. Each usually bearing only a single conidium in young cultures and occasionally a short geniculate extension with a second or third conidium. Conidiogenous sites terminal or intercalary, proliferating sympodially, brown. Occasional chain formation of two spores through secondary conidiophores on the tip or basal cells of primary conidia. Conidia mature in broad-cylindrical morphologies, usually narrow ellipsoid, ovoid, or cylindrical with rounded base and apex, as large as 18–26 × 7–10 µm, with 3(–4) thickened transverse septa and rarely one longitudinal septum, representing a high percentage of the mature population (Fig. 4A, B). Conidia occasionally forming chlamydospores during germination, exhibiting single-celled, spherical or oval brown structures on both ends of conidia, measuring 7–12 µm in diameter (Fig. 4C). Another population of conidia with 4–5(–6) transverse septa, smooth-walled, cylindrical to obclavate, mostly straight, sometimes curved with middle cells slightly enlarged, pale brown to brown, with rounded basal and apical cells, 30–36 (–38) × 7–10(–12) and 0–1(–2) longitudinal septa in 2–3 of the transverse segments. A few conidia with 7 transverse septa, usually without longitudinal septa, 35–39 × 10–11 µm, relatively narrowed cylindrical, not forming secondary conidiophores (Fig. 4D, E). Conidiophores emerging from the surface of dead infected plants rigid, brown, clustered, or scattered, with 1–4(–6) conidiogenous loci, 25–67 µm long and 3.8–6 (–7.5) μm thick (Fig. 4F). Most conidia with 8–10(–11) transverse septa, 44–55 (–59) × 8–10 µm. On aged parts of the colony, conidia usually clustered in small clumps with 2–4 spores near the tips or laterals of primary conidiophores (Fig. 4G).

All isolates producing dark-brown, compound chlamydospores, looking like thickened oval or rounded cell chains with punctate ornamentation, arranged in chains (Fig. 4H). Such cells conglomerating and elongating to a pearl necklace or an irregular shape, as large as 15–41 × 20–53 µm with 3–8 cells; these structures enlarging as the colony ages and observed also on six-week inoculated leaf debris in contact with soil. Conidia color before full maturity dark yellow, which deepens to an olive brown, paler towards apex, against which the thickness of major transverse septa contrasts strongly.

Culture characteristics after 7 days—Colonies color and aspect of the holotype strain on PDA (Fig. 4I), PCA (Fig. 4J), MEA (Fig. 4K), and OA (Fig. 4L) are provided in Table 2. This strain grows over a wide range of temperatures with varying growth rates. Among the different temperature and culture media, the best mycelial growth occurs at 25 °C on PDA and PCA. On all media, the strain grows slowly at 4 °C and 35 °C, moderately at 16 °C, rapidly between 20–30 °C, and does not grow at 40 °C.

**Table 2.** Cultural characters and temperature effect on new species growth after 7 days of incubation on PCA, PDA, MEA, and OA.

Species	Media	Colony type	Colony color	Reverse color	Pigmentation	Sporulation at 25 °C	Colony diameter (mm) at					
							4 °C	16 °C	20 °C	25 °C	30 °C	35 °C
*A.radicicola*	PCA	Velvety to glaborous	Olive brown (4F8)	Olive brown (4F7)	none	Moderate	6.6 ± 0.5	54.0 ± 0.7	74.9 ± 2.7	81.8 ± 0.7	79.1 ± 7.7	35.6 ± 7.6
	PDA	Cottony, compact	Olive brown (4E4)	Olive brown (4F5)	yellowish brown (5E5)	Moderate	5.9 ± 0.3	50.8 ± 1.0	76.1 ± 1.6	80.7 ± 2.8	78.6 ± 1.6	29.1 ± 5.3
	OA	Cottony, compact	Olive brown (4D3/4F7)	Yellowish brown (5F5)	none	Poor	5.5 ± 0.0	48.0 ± 0.8	63.2 ± 2.8	73.3 ± 1.7	67.1 ± 2.7	25.2 ± 3.1
	MEA	Cottony compact, pleated	Greyish beige (4C2/4E3)	Yellowish brown (5F6)	yellowish brown (5E5)	Poor	5.5 ± 0.4	46.8 ± 0.5	72.6 ± 1.4	78.2 ± 5.4	70.0 ± 0.8	25.8 ± 4.4
*A.longiformis*	PCA	Velvety	Olive (3E3)	Olive (3E4)	none	Abundant	5.6 ± 0.3	46.0 ± 2.0	66.9 ± 0.6	77.0 ± 0.8	54.4 ± 1.6	6.8 ± 0.3
	PDA	Cottony compact	Dull green (29E3)	Dark green (29F8)	none	Moderate	5.8 ± 0.3	44.0 ± 0.8	66.4 ± 1.1	66.5 ± 0.6	45.5 ± 1.3	6.0 ± 0.4
	OA	Cottony compact	Dull green (30D3/30E)	Dull green (30F8)	none	Poor	5.3 ± 0.3	44.8 ± 1.3	58.5 ± 1.3	67.5 ± 0.6	55.8 ± 1.9	5.4 ± 0.5
	MEA	Cottony dense	Dull green (28E3)	Olive brown (4D4)	none	Poor	5.4 ± 0.3	39.4 ± 0.5	55.5 ± 0.6	60.5 ± 0.4	46.9 ± 1.3	6.9 ± 0.3

###### Additional isolate examined.

Algeria • Oran City, Oran province Market, from the root of *Daucuscarota*. 18 February, 2020, N. Bessadat, (CBS 149906, preserved in a metabolically inactive state in the Westerdijk Fungal Biodiversity Institute, Utrecht, the Netherlands). Living culture NB794.

Algeria • Mascara City, Tizi province, from leaves of cultivated *Daucuscarota*. 21 December, 2020, N. Bessadat, Living culture NB936.

###### Notes.

Phylogenetic analyses indicated that *Alternariaradicicola* sp. nov. fell in an individual branch close to *A.tellustris* (ex-type, CBS 538.83) and *A.chlamydosporigena* (CBS 341.71). Although the three species shared identical ITS sequences, there were 8/529 differences in *gpd*, 8/833 in *rpb2*, and 8/199 in *tef1* between isolates of *A.radicicola* sp. nov. and *A.tellustris* and 8/529 differences in *gpd*, 11/833 in *rpb2*, and 7/199 in *tef1* between isolates of *A.radicicola* sp. nov. and *A.chlamydosporigena*. Although *A.radicicola* sp. nov. has almost the same conidia size and shape as the closely related *A.chlamydosporigena* (Table 3), it differs from this species by producing chlamydospores in culture that are able to form fertile conidiophores; association of conidiophores with the chlamydospores has never been observed on *A.chlamydosporigena* (Simmons 1971; Marin Felix et al. 2019). Other species from section Embellisia exhibited variable conidial size; *A.embellisia* produces chlamydospores in pairs or chains (up to seven cells) (Delgado Ortiz et al. 2019), while *A.radicicola* isolates form shorter chains of 3–4 cells after 7 days under similar incubation conditions. *Alternariatellustris* produces obclavate or long ellipsoid conidia smaller than the new species (18–33 × 6–8 vs. 20–38 × 7–12, respectively); obclavate conidia were rare or mainly immature in *A.radicicola* sp. nov. Chlamydospore production was influenced by temperature and time of incubation. *A.radicicola* sp. nov. isolates exhibited abundant chlamydospores beyond 7 days when temperatures ranged between 30–35 °C and 2–3 weeks when incubated at 20–25 °C. These structures were also observed on infected host leaves after 6 weeks of inoculation.

**Table 3.** Morphological comparison of the new species and other *Alternaria* species in sections *Embellisia* and *Embellisioides*.

Section	Species	Conidia morphology				Conidia chain	Chlamydospores	Reference
		Shape	Size (µm)	septa				
				Transverse- septa	Longitudinalsepta			
*Eureka*	*A.cumini*	Ovoid, long-ovoid or obclavate, beakless	50–90 × 13–23	6–11	1–3 (4)	3–4	Absent	Simmons 2007
	*A.eureka*	Short ovoid or broadly ellipsoid	15–30 × 12–13	4–6	1–2 per transverse segment	1–2	Absent	Simmons 1986
	*A.hungarica*	Ovoid, broadly ellipsoid with no definable beak	28–35 × 14–21	3–4 (6)	1–3 per transverse segment	2–4	Absent	Toth et al. 2011
*Embellisia*	*A.chlamydosporigena*	Subcylindrical, rounded at both ends	23–28 × 7–9	3–5 (7)	0–1 (2)	1	Abundant	Hoog and Muller 1973
	*A.embellisia*	Ellipsoidal, subcylindrical rounded at both ends	30–40 × 10–12	4–6 (10)	0–1	1	Absent to rare	Simmons 1971
	***A.radicicola* sp. nov.**	Narrow ellipsoidal, ovoid or cylindrical, straight or slightly curved	20–38 × 7–10 (12)	3–5 (7)	0–1 (2)	1	Abundant	Present study
	*A.tellustris*	Obclavate or long ellipsoid	18–33 × 6–8	2–3 (5)	0–1 (2)	1	Abundant	Gannibal and Gasich 2019
*Embellisioides*	*A.hyacinthi*	Oblong or obclavate subcylindrical, clavate, fusiform	14–39 × 8–13	2–4 (7)	1–2	1	Absent	Hoog and Muller 1973
	*A.lolii*	Ellipsoid to ovoid	45–60 × 10–18	6–13	0–1	1–3	Rare	Simmons 2004
	***A.longiformis* sp. nov.**	Oblong, ellipsoid or subcylindrical	40–80 (97) × 10–18 (25)	4–10 (15)	0–2	1–2	Absent to rare	Present study
	*A.novae–zelandiae*	Ovoid, cylindrical	28–37 × 10	5–8	3–4	1	Absent	Simmons 1990
	*A.planifunda*	Ellipsoid, ovoid with broad flat base	20–28 × 10–13	3–4	1–2 (3)	1–2	Present	Simmons 1983
	*A.proteae*	Ovoid, long ellipsoid or obovoid	18–32 × 7–12	2–6 (–7)	0–3	1	Absent	Simmons 1990
	*A.tumida*	Long ovoid, ellipsoid, straight or rarely slightly inequilateral	35–42 × 13–18	3–5	0–1 (2)	1	Abundant	Simmons 1983

#### ﻿Section Embellisioides

##### ﻿ Alternaria longiformis

Taxon classificationFungiPleosporalesPleosporaceae

N. Bessadat & P. Simoneau sp. nov.

998E4A47-1F52-531F-B338-FC9483BA250C

 848582

Fig. 5

###### Etymology.

Name refers to conidial shape and size, which is longer than other species within the section Embellisioides.

###### Type.

Algeria • Mostaganem, Hassi Mamache on infected leaves of *Solanumlycopersicum*. 22 May, 2015, N. Bessadat, (INH001055, holotype), preserved in a metabolically inactive state via deep freezing at INH herbarium, France, using the COMIC technical platform, ex-type culture (CBS149901, NB354).

###### Description.

**Figure 5.**
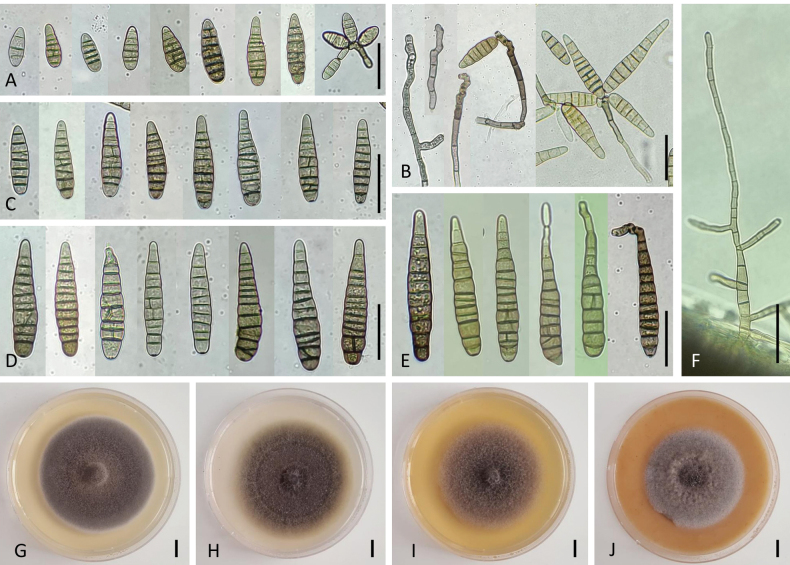
Morphology of *Alternarialongiformis* sp. nov. from *Daucuscarota*: Conidia on PCA for 7 days at 22 °C (**A–E**); Conidiophores and sporulation patterns on PCA for 7 days at 22 °C (**B**); Conidiophores on inoculated carrot leaves at 21 JPI (**F**); Colony phenotypes on PDA (**G**), PCA (**H**), MEA (**I**), and OA (**J**) for 7 days at 25 °C. Scale bar: 25 µm (**A–F**); 10 mm (**G–J**).

On PCA, attaining 69 mm diam., colony wooly, loose, with aerial branched subhyaline hyphae and 2–3 pairs of moderately defined concentric rings of growth and sporulation. During an initial 5–7 d of growth, colony producing only minor sporulation near the agar surface. At the same time, abundant, long, suberected aerial hyphae arising throughout light-deprived parts of the colony. The tip and some branches of these slender aerial axes enlarging into well-defined conidiophores with few lateral branches, mostly near the hyphal apex, from a simple and short conidiophore bear 2–4 conidia (Fig. 5A, B), yielding to an open layer of sporulation in the surface of the colony. Sporulation pattern forming compact, small clumps of conidia. Conidiophores septate, simple or sparingly branched, straight to slightly curved, pale to medium brown, with series of 2–6(–14), geniculate, sympodial conidiogenous sites. Primary conidiophores of short length, 15–20 × 4–7 µm, cylindrical, 0–3-septate, produced from fertile hyphae, commonly becoming 35–80 (–170) µm long with 4–9 transverse septa. Each conidiophore bearing a single conidium, rarely a chain of 2 conidia in undisturbed young colonies. Conidiogenous cells terminal or intercalary, solitary or proliferating sympodially (Fig. 5B). Seldom production of secondary conidiophores from primary conidia forming short 5–23 (–41) × 3.5–7.5 µm, 0–3-septate, cylindrical or angular complexity structure at the apex leading to false conidia chains upon the aging parts of the colony. Mature conidia with 4–7 transverse septa, 31–60 × 10–18 µm, oblong, ellipsoid, or subcylindrical, broadly rounded at the base with a bluntly rounded apical cell (Fig. 5C, D). Conidia at full development tapering gradually from narrowly ovoid into narrowly cylindrical, 56–90 (–100) × 12.5–17 µm, always rounded at base tapering towards apex with 8–11 (–15) transverse septa (Fig. 5E). A few submedian cells increasing in width and producing thin longitudinal and oblique septa. Abundant juvenile conidia, 5–20 × 2.5–12.5 µm, spherical to ovoid at the center of the colony at 14d, formed through extension of secondary conidiophores and usually 1–2-celled. Fully developed conidia mostly equilateral until enlargement of a few body cells and their secondary internal septation cells introducing minor degree of curvature. Conidia slightly, to distinctly constricting at their 1–3 transverse septa, contrastingly darker than others. One to two longitudinal septa very pale inserted in 1–3 first transverse segments. Conidia color pale to medium yellow, appearing quite smooth, thin-walled due to a lack of ornamentation on the surface. Conidium germination after 24 h usually bipolar but not conspicuous from a non-polar cell. Conidiophores emerging from the surface of dead infected plants brown, scattered, with 1–3 (–6) conidiogenous loci, 25–67 (–100) µm long and 3.8–6 (–7.5) μm thick (Fig. 5F). Most conidia with 8–10 (–11) transverse septa, 44–55 (–59) × 8–10 µm.

Culture characteristics after 7 days— Colonies color and aspect the holotype strain on PDA (Fig. 5G), PCA (Fig. 5H), MEA (Fig. 5I), and OA (Fig. 5J) are provided in Table 2. This strain grows over a wide range of temperatures with varying growth rates. Among the different temperature and culture media, the best mycelial growth occurs between 20–25 °C on PDA. On all media, the strain grows slowly below 4 °C, moderately at 16 °C and 30 °C, rapidly between 20–25 °C, and does not grow well at a temperature of 35 °C. Mycelial growth was inhibited at 40 °C.

###### Additional isolate examined.

Algeria • Mascara City, Tizi province, from leaves of *Daucuscarota*. 21 December, 2020, N. Bessadat, (CBS 149905, preserved in a metabolically inactive state in the Westerdijk Fungal Biodiversity Institute, Utrecht, the Netherlands). Living culture NB930.

###### Notes.

Phylogenetic analyses indicated that *Alternarialongiformis* sp. nov. fell in an individual branch close to *A.lolii* (ex-type, CBS 115266). Between this species and *A.lolii*, there were 4/464 differences in ITS, 3/529 in *gpd*, 15/833 in *rpb2*, and 8/199 in *tef1*. Isolates of *Alternarialongiformis* sp. nov. are morphologically similar to *A.lolii* (Bessadat et al. 2021). Conidia of both species are quite similar in shape but different in size (Table 3), and primary conidiophores are geniculate but slightly different in size (25–150 × 3–5 in *A.lolii* and 35–170 × 4–7 μm in *A.longiformis*). These two species can be distinguished mainly by their sporulation patterns, the presence/absence of cellular knots, and the abundance of secondary conidiophores. *Alternarialolii* was reported to produce distinctive submerged knots of hyphal cells and emergent rhizoidal branches (Simmons 2004). This species produces rarely chlamydospores (Table 3). None of these structures were observed on the two *A.longiformis* sp. nov. isolates. Further, branching through secondary conidiophores is conspicuous in *A.lolii*, which is not in *A.longiformis* sp. nov. Other species belonging to the same section, such as *A.proteae*, *A.novae–zelandiae*, and *A.hyacinthi*, form multi-geniculate conidiophores (Hoog and Muller 1973; Simmons 1990) and shorter conidia compared to *A.longiformis* sp. nov. (Table 3). *Alternariaplanifunda* and *A.tumida* were reported to produce conspicuous chlamydospores and smaller, solitary conidia (Simmons 1983), making them distinct from the new species (20–28 × 10–13 and 35–42 × 13–18 vs. 40–97 × 10–25, respectively).

### ﻿Pathogenicity tests

To assess the host range of *Embellisia*-like isolates, three species from Apiaceae, i.e., carrot (*Daucuscarota*) var. muscade, coriander (*Coriandrumsativum*), and fennel (*Foeniculumvulgare*) var. dulce, were inoculated with seven isolates collected on Apiaceae and one from Solanaceae. Two reference strains from sections *Eureka* (*A.hungarica*CBS 123925) and *Radicina* (*A.petroselini*CBS 109383) were used for comparison.

**Figure 6.**
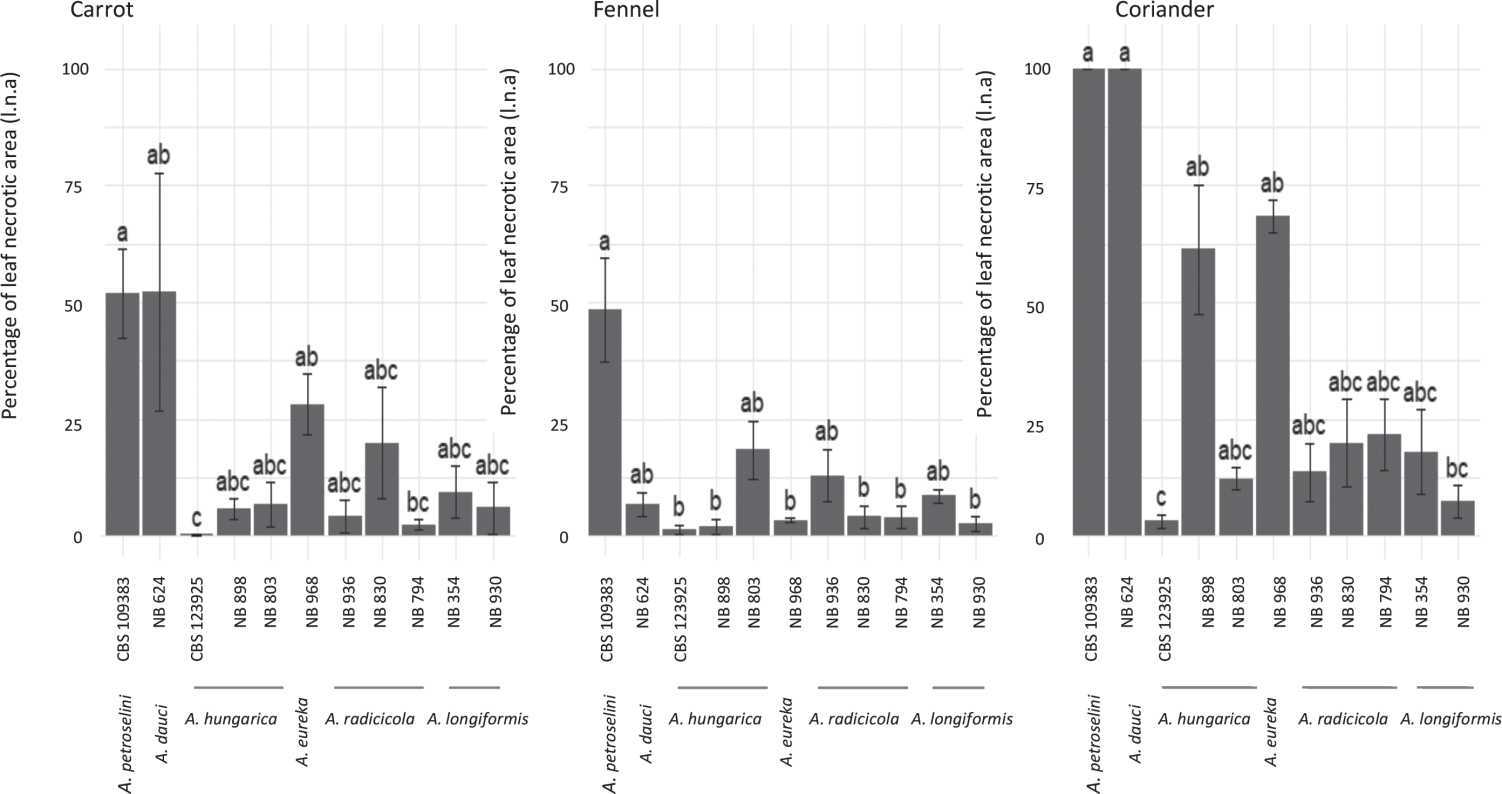
Percentage of leaf necrotic area recorded at 14 DAI on eight-week-old Apiaceae plants inoculated with eight *Embellisia*-like isolates plus two reference strains (*A.petroselini*CBS 109383, *A.hungarica*CBS 123925).

ANOVA of l.n.a. averages and standard error of the triplicates at day 14 using the data of the first three leaves was calculated (Fig. 6).

**Figure 7.**
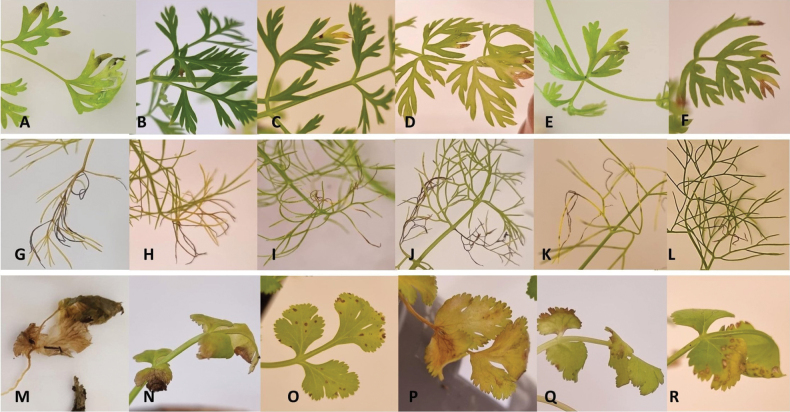
Symptoms developed on leaves of *Daucuscarota* (**A–F**), *Foeniculumvulgare* (**G–L**), and *Coriandrumsativum* (**M–R**) inoculated with *Alternaria* strains: *A.petroselini* (CBS 109383) (**A, G, M**), *A.hungarica* (NB898) (**B, H, N**) and (NB803) (**C, I, O**), *A.eureka* (NB968) (**D, J, P**), *A.radicicola* sp. nov. (NB830) (**E, K, Q**), and *A.longiformis* sp. nov. (NB930) (**F, L, R**), after 21 days.

*Alternariapetroselini* (CBS 109383) was classified as highly aggressive on all tested plant species, with an average l.n.a. above 50% and even reaching 100% on coriander. On this plant species, this isolate produced severe blighted areas on inoculated leaves and petioles at 14 dai, which coalesced to encompass the entire leaves at 21 dai (Fig. 7M). By contrast, irrespective of the plant species, *A.hungarica* CBS123925 was poorly aggressive. Minor necrotic symptoms were observed on carrot (0.2%) and fennel (0.8%) inoculated with this strain as well as with other tested isolates of this species, NB898 (3.5–1.5%, respectively) (Fig. 7B, H) and NB803 (4.2–13.8%, respectively) (Fig. 7C, I). *Alternariaeureka* isolate NB968 was moderately pathogenic on carrot (28.1%) while poorly aggressive on fennel with an average l.n.a. of 2.4% (Figs 7D, J). Brown lesions were observed on tips of basal carrot and fennel leaves, which mainly did not expand when inoculated with *A.radicicola* sp. nov. isolates NB830 (12–2.9%, respectively) (Fig. 7E, K), NB936 (3.1–7.8%, respectively), and NB794 (1.6–1.9%, respectively). Similarly, *A.longiformis* sp. nov. was also weakly pathogenic on carrot and fennel; minor symptoms were provoked with isolates NB930 (3.6–1.5%, respectively) (Fig. 7F, O, L) and NB354 (5.9–6.1%, respectively), and lesions did not expand at 21 dai. Although not significantly different, the percentage of l.n.a on coriander was slightly higher with two isolates from section Eureka NB898 (61.8%) and NB968 (68.4%). Other isolates of the same section (NB803 and CBS 123925) were weakly pathogenic on coriander (12.2% and 1.9%, respectively). Isolates representing new species from section Embellisia (NB936, NB830, and NB794) caused mild symptoms (13.7–19.9% and 22.2%, respectively), including brown spots and chlorosis on the lower leaves of coriander at 21 dai (Fig. 7Q). Isolates of section Embellisioides (NB930 and NB354) were also weakly pathogenic on coriander (7.3–16.8%, respectively), forming light brown necrosis on the edge of basal leaves after 21 dai (Fig. 7R). No symptoms were seen in the negative control plants, and re-isolatedfungi formed similar conidia on PCA and inoculated leaves.

## ﻿Discussion

In surveys to assess the diversity of *Alternaria* spp. from northwest Algeria regions, hundreds of isolates were sampled from wild and cultivated Apiaceae. Although they represented a minor portion of this collection, studied isolates revealed a high diversity. Morphological observations indicated that the seven selected isolates were members of three different sections, *Embellisia*, *Embellisioides*, and *Eureka*, according to previous descriptions of conidia shape and size (Simmons 1971, 1983, 1986, 1990, 2007; Toth et al. 2011; Woudenberg et al. 2013). It is important to note that these species do indeed possess morphological similarities in the form of thick, dark, and rigid septa. However, these key features should not be overlooked for a valid taxonomic distinction of closely related species (Woudenberg et al. 2013). The multilocus phylogenetic analysis based on four loci, ITS, *gpd*, *tef1*, and *rpb2*, showed that they were members of four distinct species, two of them being newly described species for the genus identified as *A.radicicola* and *A.longiformis* spp. nov. The main difference between these new species and isolates of section Eureka is that conidia are elliptic, elongated, or nearly cylindrical, sometimes slightly curved (asymmetrical), brown, with thick transverse septa darker than the outer wall of conidia.

These two species grouped isolates that were closely related to but significantly separated from *A.lolii* in section Embellisioides and from *A.chlamydosporigena* and *A.tellustris* in section Embellisia. Section Embellisia now includes four species, while section Embellisioides includes seven species. This constitutes the first description of new species in these two sections since their definition by Woudenberg et al. (2013).

Isolates from section Eureka exhibited high phylogenetic and morphological variations and clustered as two different taxa identified as *A.eureka* and *A.hungarica*.

Some *Embellisia*-likefungi are considered plant pathogens (Delgado Ortiz et al. 2019), while others are known to occur in seawater, soil, and in relatively extreme environments (Simmons 1983, 1990; David et al. 2000). *Alternariaeureka* was isolated from *Cladanthusarabicus* (Ebrahim et al. 2013), deciduous holly (Basson et al. 2019), canola, and other Brassicaceae (Al-lami et al. 2019). This species was also recorded on *Triglochinprocera*, *Medicagorugosa* (Simmons 1986; Woudenberg et al. 2013), and *Dactylisglomerata* (Sanchez Marquez et al. 2007). These two later hosts are common weeds that were frequently observed on carrot fields. Here we report wild carrot as a natural host of *A.eureka*. Pathogenicity tests confirmed the ability of this species to provoke necrotic lesions on carrot leaves and other apiaceous plants under greenhouse conditions. In the same vein, new host records of *A.hungarica* on cultivated and wild *Daucuscarota* in growing fields were provided. This species was originally isolated and described as a minor foliar pathogen for wheat in Hungary (Toth et al. 2011), and to our knowledge, no additional records of this species have been reported. Isolates of this species were weakly pathogenic on all apiaceous plants tested in the present study. On the other hand, isolates representing the two new species, viz., *A.radicicola* and *A.longiformis*, were able to infect carrot, coriander, and fennel and induce small necrotic lesions on basal leaves after 21 dai. Therefore, these species, along with *A.eureka* and *A.hungarica*, may simply occupy various ecological niches, and their host plants may serve as a source of secondary inoculum, causing asymptomatic infections on several cultivated crops.

In conclusion, this study constitutes the first record of *A.eureka* and *A.hungarica* in Algeria. Detection of these two species, along with two newly described ones, confirms previous observation of a huge diversity of *Alternaria* spp. in Algeria (Bessadat et al. 2021). This might be due to favorable climatic conditions but also to non-appropriate cultural practices. For example, in the last several years, growers were used to leaving diseased plant tissues and weeds in the field after harvesting the crop. Such conditions may constitute a source of inoculum for infection initiation and continuation throughout the growing season in many crop systems (Lin and Hand 2019). Furthermore, climate change, such as increases in temperature events and dust storms, can aerosolize fungal spores and primefungi to adapt to previously inhospitable environments (Seidel et al. 2024). Additional studies on the new species are necessary to elucidate their host range, distribution, as well as their specificity.

## Supplementary Material

XML Treatment for Alternaria eureka

XML Treatment for Alternaria hungarica

XML Treatment for Alternaria radicicola

XML Treatment for Alternaria longiformis

## ﻿Additional information

### Author ORCIDs

Nabahat Bessadat https://orcid.org/0000-0001-7795-2606

Nelly Bataillé-Simoneau https://orcid.org/0000-0001-6200-4259

Justine Colou https://orcid.org/0000-0002-9623-4412

Bruno Hamon https://orcid.org/0000-0001-5868-0676

Kihal Mabrouk https://orcid.org/0000-0003-2901-373X

Philippe Simoneau https://orcid.org/0000-0002-3890-9848

### Data availability

All of the data that support the findings of this study are available in the main text or Supplementary Information.

## Supplementary materials

Supplementary material 1 Phylogenetic tree Data type docx https://binary.pensoft.net/file/1241728This dataset is made available under the Open Database License (http://opendatacommons.org/licenses/odbl/1.0/). The Open Database License (ODbL) is a license agreement intended to allow users to freely share, modify, and use this Dataset while maintaining this same freedom for others, provided that the original source and author(s) are credited.Nabahat Bessadat, Nelly Bataillé-Simoneau, Justine Colou, Bruno Hamon, Kihal Mabrouk, Philippe Simoneau
