# Supplementary material for: ﻿New members of Alternaria (Pleosporales, Pleosporaceae) collected from Apiaceae in Algeria
Source: MycoKeys. 2025 Feb 4;113:169–92. doi: 10.3897/mycokeys.113.138005 (PMC11815330; doi:10.3897/mycokeys.113.138005)
Supplement: Supplementary material 1 — Phylogenetic tree [file mycokeys-113-169-s001.docx]

**Figure S1.** Phylogenetic tree reconstructed by the maximum likelihood method from the alignment of *gpd* sequence*s* of *Embellisia-*like isolates of *Alternaria* from Apiaceae and of *Alternaria* species representing the 29 sections of the genus.
